# A User Association and Resource Allocation Algorithm for UAV-Assisted Smart Grid

**DOI:** 10.3390/s24248195

**Published:** 2024-12-22

**Authors:** Jianwei Wei, Yuzhu Lei, Zhiyi Wen, Yongqing Xiao, Pengcheng Ma, Lingtao Sun, Lin Su

**Affiliations:** 1 Xuejiawan Power Supply Company, Ordos 010300, China; jianweiwei61@gmail.com (J.W.); xgwzy@163.com (Z.W.); yongqing_xiao333@163.com (Y.X.); pengchengma202412@163.com (P.M.); lingtao_sun@163.com (L.S.); linsu8877@163.com (L.S.); 2 College of Information Science and Electronic Engineering, Zhejiang University, Hangzhou 310027, China

**Keywords:** UAV, user association, resource allocation, smart grid, heterogeneous wireless network

## Abstract

Recently, massive intelligent applications have emerged for the smart grid (SG), such as inspection and sensing. To support these applications, there have been high requirements on wireless communication for the SG, especially in remote areas. To tackle these challenges, a UAV-assisted heterogeneous wireless network is proposed in this paper for the SG, where multiple UAVs and a macro base station collaboratively provide a wide range of communication services. To further improve the communication capacity of this system, a joint user association and resource allocation algorithm is developed to maximize the total system throughput. To solve this problem, a matching algorithm is first proposed to solve the user association and subchannel assignment optimization problem. Then, the Lagrangian dual method is utilized to solve the power allocation problem. Finally, extensive simulations show that the proposed algorithm can effectively increase the user communication rate and enhance the capacity of the heterogeneous network for the SG.

## 1. Introduction

**Figure 1:**
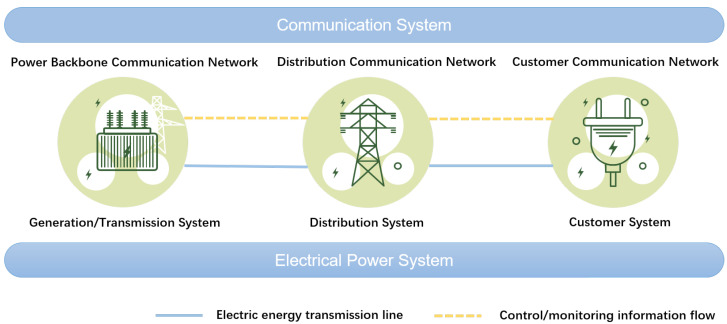
The SG communication system architecture.

Recently, the smart grid (SG) has emerged, breaking through the traditional mode of raw power supply and consumption [1]. Utilizing advanced information, control, and communication technologies, the SG provides technical support for safe and efficient use of electricity with reduced energy consumption and greenhouse gas emissions [2,3]. To realize the above functions, an effective, reliable and robust communication system is indispensable [4,5]. As shown in Figure 1, the communication network of the SG can be divided into a power backbone communication network, distribution communication network, and customer communication network [6,7].

The power backbone communication network connects high-voltage power plants and substations, and it is dominated by optical fiber communication. The customer communication network refers to the communication network formed by the interconnection of household electricity equipment. In contrast, the distribution communication network covers medium- and low-voltage power plants, substations, distributed power sources, and other facilities. It carries out information interaction between the distribution side and the upper control center and supports distributive automation, distributive operation monitoring, power consumption information collection, and other services [8,9,10]. Therefore, the SG is directly oriented to electricity customers and society. With wide coverage, a large number of devices and complex communication services, the communication system occupies a pivotal position in the SG and has high requirements for security and reliability. For the complex and diverse communication demands, multiple communication networks complement each other and form heterogeneous networks to realize high-speed seamless coverage in the SG [11,12]. Moreover, to satisfy the power demand of users, some terminal devices are distributed in remote areas with complex terrain and poor communication conditions [13]. These impose strict requirements on the network planning and user association of the SG. As to the above challenges, unmanned aerial vehicles (UAVs) are able to provide auxiliary communication services due to the characteristics of low cost, high mobility, and self-adaptive capability [14,15,16]. Therefore, we consider the UAV-assisted heterogeneous wireless network (HWN) for the SG in remote areas, where UAVs and a macro base station (BS) reuse spectrum resources to supplement communication coverage for a large number of power terminals, such as sensors, controllers, and cameras. Note that the UAV-assisted communication is still a relatively low-cost and reliable method in most normal scenarios. In extreme scenarios, such as extreme weather, satellite communication and other methods can be used instead, which is outside the scope of our work.

The user association has always been the most fundamental and critical problem in the HWN [17,18,19]. Generally, the user association algorithms include the maximum signal-to-interference-to-noise ratio (SINR) algorithm, multi-attribute decision-making (MADM) algorithm, game theory-based algorithm, and utility-based algorithm [20,21,22]. In the max-SINR algorithm, the user simply associates with the BS with the highest SINR. Differently, the MADM algorithm considers multiple attributes of networks and users, including user preferences, business requirements, network differences, etc. [23,24]. The weights of each attribute are determined through methods such as hierarchical analysis [24] and fuzzy logic, and then the weighted attributes are combined to select the network with the best comprehensive performance. Meanwhile, the game theory-based method considers the impact between user behavior and network behavior, and it finds a compromise solution to balance the interests of users and operators [25,26,27]. For example, the authors in [27] have proposed an algorithm that determines the access scheme through a non-cooperative game in multiple network overlapping areas, and the system finally reaches game equilibrium. Moreover, the utility-based algorithm optimizes resource allocation and network performance by proposing utility functions to quantify the benefits of utilizing different resources [28,29]. However, the above works may lead to load imbalance and low resource utilization due to differences in BSs in HWNs. In addition, the existing algorithms such as MADM have a high complexity, which would increase the workload of communication networks. Nevertheless, the utility-based algorithm is the preferred choice, with relatively low design and computational complexity, as well as favorable performance.

As mentioned earlier, the communication environment of the SG is complex and has a large coverage in general, which leads to a crucial requirement on a flexible communication network. In addition, a more efficient algorithm with low complexity is needed to make such a system more practical. Therefore, in this paper, we propose a user association and resource allocation algorithm for a UAV-assisted HWN in the SG, which maximizes the system throughput while ensuring the quality of service (QoS) of each user. The main contributions are summarized as follows:

- UAV-assisted heterogeneous network architecture for the SG: A UAV-assisted heterogeneous network framework is proposed for SG communication scenarios. The power terminal can flexibly select the BSs equipped on UAVs or macro BSs for communication. The framework can meet the communication demand in remote areas without sufficient communication coverage.
- Joint user association and resource allocation optimization algorithm: To further improve the system capacity of the SG, we formulate the optimization problem to jointly optimize the user association, subchannel allocation and power allocation. To efficiently solve this problem, a joint optimization method based on matching algorithm and Lagrangian dual algorithm is proposed. Therein, based on the proposed user-side and BS-side utility functions, the heuristic algorithm can obtain suboptimal solutions with low complexity.

The framework and algorithms described above can enhance the performance of SG communications. On one hand, UAVs can provide a wider range of communication coverage, ensuring the power supply in remote areas. On the other hand, the proposed joint optimization algorithms can further enhance system capacity, allowing the system to adjust the selection of BSs and resource allocation based on channel conditions.

The remainder of this paper is arranged as follows. In Section 2, we introduce the system model of the UAV-assisted HWN. Then, the problem formulation of maximizing system throughput is analyzed. In Section 3, the proposed joint user association and resource allocation algorithm is described in detail. In Section 4, experimental findings are provided to validate the proposed algorithm. This paper is finally concluded in Section 5.

## 2. System Model and Problem Formulation

### 2.1. System Model

**Figure 2:**
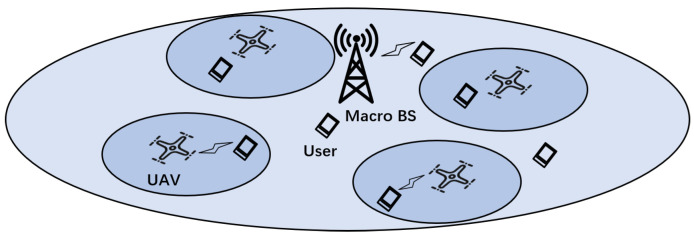
UAV-assisted HWN in the SG.

Figure 2 shows the proposed UAV-assisted HWN in the SG. In the scenario, macro BS and micro BSs equipped on the multiple UAVs coexist with overlapping coverage areas to provide communication services for randomly distributed power terminals, such as sensors, controllers, and cameras. To improve the spectrum utilization, the system uses orthogonal frequency division multiple access (OFDMA) for communication. Macro BS and *M* micro BSs share a set of orthogonal subchannels S={1,2,…,N}. The *N* users of the macro BS occupy the *N* subchannels. For ease of analysis, we assume that there is no mutual interference between the subchannels. In addition, it is assumed that both the macro BS and the micro BSs can obtain the channel state information (CSI) of all users. For convenience of representation, we use M={1,2,…,M} to denote the set of micro BSs and K={1,2,…,K} to denote the set of users of micro BSs. In addition, we denote the set of users associated with the micro BS *m* by $K_{m}$, and denote the set of users communicating with micro BS *m* on subchannel *n* by $K_{mn}$.

### 2.2. Problem Formulation

According to Shannon’s formula, when user *k* associates with BS *m* on subchannel *n*, the communication rate can be expressed as

(1) $$R_{k,m,n}=B_{n}\log_{2}\left(1+\gamma_{k,m,n}\right),$$

where $B_{n}$ represents the bandwidth of subchannel *n*, and $\gamma_{k,m,n}$ denotes the received SINR, which can be calculated as follows,

(2) $$\gamma_{k,m,n}=\frac{p_{k,n}h_{k,m,n}}{\sum_{j\in K_{n},j\neq k}p_{j,n}h_{j,m,n}+p_{l,n}h_{l,m,n}+\sigma^{2}},$$

where $p_{k,n}$, $p_{j,n}$, and $p_{l,n}$ represent the signal power of micro BS user *k*, micro BS user *j*, and macro BS user *l* that communicate on subchannel *n*, respectively, $h_{k,m,n}$, $h_{j,m,n}$, and $h_{l,m,n}$ represent the channel gains of micro BS user *k*, micro BS user *j* and macro BS user *l* to BS *m*, respectively, and $\sigma^{2}$ represents the noise power.

To meet the communication demands of users in the SG, a minimum communication rate is set for each user, which can be expressed as

(3) $$\sum_{m\in M}\sum_{n\in S}R_{k,m,n}\geq R_{k}^{min},$$

where $R_{k}^{min}$ represents the minimum communication rate required by user *k*.

Additionally, we define a binary variable $x_{k,m}$ to represent the connection between user *k* and BS *m*. Specifically, $x_{k,m}=1$ when user *k* associates with BS *m*, and $x_{k,m}=0$ otherwise. Considering that a user can only associate with one BS at the same time, there is the following constraint

(4) $$\sum_{m\in M}x_{k,m}\leq 1,\forall k\in K.$$

Similarly, define a binary variable $y_{k,m,n}$ to represent the subchannel assignment of BS *m*. Specifically, $y_{k,m,n}=1$ when the subchannel *n* of BS *m* is occupied by user *k*, and $y_{k,m,n}=0$ otherwise. Considering that a subchannel of a BS can only be occupied by one user at the same time, and a user can only occupy a subchannel of a BS at the same time, there are the following constraints:

(5) $$\sum_{k\in K}y_{k,m,n}\leq 1,\forall m\in M,n\in S,$$

(6) $$\sum_{n\in S}y_{k,m,n}\leq 1,\forall k\in K,m\in M.$$

Considering that the communication resources of BSs are limited, when the load of each BS is relatively balanced, the entire communication system can provide more reliable services. Therefore, the load of the micro BS is limited to avoid excessive users associating with a BS at the same time, which can be given by

(7) $$\sum_{k\in K}x_{k,m}\leq q_{m},\forall m\in M,$$

where $q_{m}$ represents the maximum number of users allowed to associate with BS *m*.

In addition, due to the scarcity of spectrum resources, we consider that the macro BS and micro BSs share the same set of subchannels. However, this will lead to inter-layer interference and intra-layer interference in the HWN. In order to ensure the communication quality of macro BS users, it is necessary to ensure that the interference to macro BS users does not exceed a certain threshold. Therefore, the following constraint is imposed on the interference:

(8) $$\sum_{m\in M}\sum_{k\in K}y_{k,m,n}p_{k,n}h_{k,0,n}\leq I_{0,n}^{th},\forall n\in S,$$

where $h_{k,0,n}$ represents the channel gain from user *k* to macro BS on subchannel *n*, and $I_{0,n}^{th}$ represents the maximum interference threshold allowed for macro BS on subchannel *n*.

Finally, due to the existence of interference, it is necessary to limit the signal transmission power within the system. Therefore, there is the following constraint:

(9) $$p_{k}^{\min}\leq p_{k,n}\leq p_{k}^{\max},\forall k\in K,n\in S,$$

where $p_{k}^{\min}$ and $p_{k}^{\max}$ represent the minimum and maximum transmission power of user *k*, respectively.

For ease of representation, we denote $X=\{x_{k,m},\forall k,m\}$ as the set of user association variables, $Y=\{y_{k,m,n},\forall k,m,n\}$ as the set of subchannel assignment variables, and $P=\{p_{k,n},\forall k,n\}$ as the set of power allocation variables. By jointly considering user association, subchannel assignment, and power allocation, the following optimization problem in the UAV-assisted SG is formulated to maximize the system throughput

(10) $$\begin{array}{cc} P1\; & \max_{X,Y,P}\;\sum_{m\in M}\sum_{k\in K}\sum_{n\in S}x_{k,m}y_{k,m,n}R_{k,m,n},\\ s.t.\; & \sum_{m\in M}\sum_{n\in S}R_{k,m,n}\geq R_{k}^{min},\forall k\in K,\\ \; & \sum_{m\in M}\sum_{k\in K}y_{k,m,n}p_{k,n}h_{k,0,n}\leq I_{0,n}^{th},\forall n\in S,\\ \; & \sum_{m\in M}x_{k,m}\leq 1,\forall k\in K,\\ \; & \sum_{k\in K}x_{k,m}\leq q_{m},\forall m\in M,\\ \; & \sum_{k\in K}y_{k,m,n}\leq 1,\forall m\in M,n\in S,\\ \; & \sum_{n\in S}y_{k,m,n}\leq 1,\forall k\in K,m\in M,\\ \; & x_{k,m}\in \left\{0,1\right\},\forall k\in K,m\in M,\\ \; & y_{k,m,n}\in \left\{0,1\right\},\forall k\in K,m\in M,n\in S,\\ \; & p_{k}^{\min}\leq p_{k,n}\leq p_{k}^{\max},\forall k\in K,n\in S. \end{array}$$

## 3. Joint User Association and Resource Allocation Algorithm

It can be found the optimization problem (10) is a mixed integer nonlinear programming (MINLP) problem and cannot be solved directly. Therefore, we adopt the block coordinate descent (BCD) algorithm to decompose the optimization problem into three subproblems, i.e., user association problem, subchannel assignment problem, and power allocation problem.

### 3.1. User Association Optimization

When the subchannel assignment Y and power allocation P are given, the user association optimization problem can be expressed as

(11) $$\begin{array}{cc} P1-\mathrm{UA}\; & \max_{X}\;\sum_{m\in M}\sum_{k\in K}x_{k,m}R_{k,m,n},\\ s.t.\; & \sum_{m\in M}x_{k,m}\leq 1,\forall k\in K,\\ \; & \sum_{k\in K}x_{k,m}\leq q_{m},\forall m\in M,\\ \; & x_{k,m}\in \left\{0,1\right\},\forall k\in K,m\in M. \end{array}$$

The matching algorithm can be utilized to solve the optimization problem (11) [30,31,32]. Define $u_{UA}^{k}\left(m\right)$ as the utility function of user *k* to BS *m* and $u_{UA}^{m}\left(k\right)$ as the utility function of BS *m* to user *k*. For user *k*, if $u_{UA}^{k}\left(m_{1}\right)>u_{UA}^{k}\left(m_{2}\right)$, it is more appropriate to associate with BS $m_{1}$ than BS $m_{2}$. For BS *m*, if $u_{UA}^{m}\left(k_{1}\right)>u_{UA}^{m}\left(k_{2}\right)$, the benefit of associating with user $k_{1}$ is greater than that of associating with user $k_{2}$.

User-side utility function: We use the average SINR of all subchannels as the utility function for it, which is one of the most commonly used association judgments in user association problems [21]. Therefore, the utility function of user *k* to BS *m* on $S_{m}$ subchannels can be expressed as

(12) $$u_{UA}^{k}\left(m\right)=\log_{2}\left(1+\sum_{n\in S_{m}}\gamma_{k,m,n}\right),$$

where $S_{m}$ represents the set of available subchannels of BS *m*.

BS-side utility function: For BS *m*, to maximize the system throughput, it is profitable to associate with the user with a higher communication rate. Therefore, the utility function of BS *m* to user *k* on $S_{m}$ subchannels can be expressed as:

(13) $$u_{UA}^{m}\left(k\right)=\sum_{n\in S_{m}}p_{k,m}h_{k,m,n}.$$

Let $PL_{UA}^{k}$ denote the preferences list of user *k* to BSs, where BSs are sorted in descending order of their utility functions. Similarly, set $PL_{UA}^{m}$ to be the preferences list of BS *m* to users. According to the preference list, each user will send a connection request to the BS that can enable it to achieve a higher rate. Subsequently, the BS that receives the request will decide whether to accept the connection request based on its preference list and remaining capacity. The specific execution process is described in Algorithm 1.
**Algorithm 1 **User association based on matching algorithm.**Initialization**: $K,M,K^{rej}=K,K_{m}^{req}=K_{m}^{rej}=\emptyset ,q_{m}$.Each BS *m* broadcasts its available subchannel $S_{m}$.Each user *k* generates its preference list $PL_{UA}^{k}$.**while **$K^{rej}\neq \emptyset$** do**   Update $K^{rej}\leftarrow K,K_{m}^{req}\leftarrow \emptyset$   **for** k∈K **do**     Find $m=\arg\max_{m\in PL_{UA}^{k}}u_{UA}^{k}\left(m\right)$.     Update $K_{m}^{req}\leftarrow K_{m}^{req}\cup \left\{k\right\}$.   **end for**   **for** m∈M **do**     BS *m* generates its preference list $PL_{UA}^{m}$.     **if** $∥K_{m}^{req}∥\leq q_{m}$ **then**        Update $K_{m}\leftarrow K_{m}^{req}$.     **else**        **repeat**          Find user $k\in K_{m}^{req}$ top-ranked in $PL_{UA}^{m}$.          Update $K_{m}\leftarrow K_{m}\cup \left\{k\right\}$.          Delete *k* from $PL_{UA}^{m}$.        **until** $∥K_{m}∥=q_{m}$     **end if**     Update $K_{m}^{rej}\leftarrow K_{m}^{req}-K_{m}$.     User $k\in K_{m}^{rej}$ removes BS *m* from $PL_{UA}^{k}$.     Update $K^{rej}\leftarrow K^{rej}-K_{m}$   **end for****end while**

### 3.2. Subchannel Assignment Optimization

When the user association X and power allocation P are given, the subchannel assignment optimization problem can be expressed as

(14) $$\begin{array}{cc} P1-\mathrm{RA}\; & \max_{Y}\;\sum_{m\in M}\sum_{k\in K}\sum_{n\in S}y_{k,m,n}R_{k,m,n},\\ s.t.\; & \sum_{k\in K}y_{k,m,n}\leq 1,\forall m\in M,n\in S,\\ \; & \sum_{n\in S}y_{k,m,n}\leq 1,\forall k\in K,m\in M,\\ \; & y_{k,m,n}\in \left\{0,1\right\},\forall k\in K,m\in M,n\in S. \end{array}$$

Obviously, the optimization problem (14) is still an NP-hard problem and cannot be solved directly. Nevertheless, the interference between BSs is fixed since P is given. Therefore, each BS only needs to consider assigning subchannels to the users associated with it. Accordingly, the subchannel assignment problem of each BS can be regarded as an independent subproblem, i.e., the optimization problem (14) can be split into *M* independent subproblems, each of which can be expressed as

(15) $$\begin{array}{cc} P1-{\mathrm{RA}}_{m}\; & \max_{Y}\;\sum_{k\in K_{m}}\sum_{n\in S}y_{k,m,n}R_{k,m,n},\\ s.t.\; & \sum_{k\in K_{m}}y_{k,m,n}\leq 1,n\in S,\\ \; & \sum_{n\in S}y_{k,m,n}\leq 1,\forall k\in K_{m},\\ \; & y_{k,m,n}\in \left\{0,1\right\},\forall k\in K_{m},n\in S. \end{array}$$

Similar to the solution of the user association optimization problem, we also utilize the matching algorithm to solve the subchannel assignment problem. Define $u_{RA}^{mk}\left(n\right)$ as the utility function on the user side to represent the preference of user *k* associated with BS *m* to subchannel *n*, and define $u_{RA}^{mn}\left(k\right)$ as the utility function on the subchannel side to represent the preference of subchannel *n* to user *k*. The specific definitions are as follows.

User-side utility function: When user *k* associates with BS *m*, the utility function of the user on subchannel *n* is defined as

(16) $$u_{RA}^{mk}\left(n\right)=R_{k,m,n}.$$

which means users will occupy the subchannel with a higher rate.

Subchannel-side utility function: When user *k* associates with BS *m*, the utility function for evaluating the user on the subchannel side is defined as

(17) $$u_{RA}^{mn}\left(k\right)=\alpha \frac{R_{k,m,n}-R_{k}^{min}}{R_{k,m,n}}-C_{k,n},$$

where α is the weight coefficient, and $C_{k,n}$ represents the interference aroused by user *k* on subchannel *n*, which can be expressed as

(18) $$C_{k,n}=\beta \;p_{k,n}h_{k,0,n}+\gamma \sum_{j\in M,j\neq m}p_{k,n}h_{k,j,n},$$

where β and γ are the weight coefficients. According to (17), it can be found that BSs always allocate subchannels to users who can increase the communication rate while causing less interference to other BSs.

Denote $PL_{RA}^{mk}$ as the preferences list of user $k\in K_{m}$ to available subchannels of BS *m*, where subchannels are sorted in descending order of their utility functions. Similarly, set $PL_{RA}^{mn}$ to be the preferences list of subchannel *n* to users associated with BS *m*.

The proposed subchannel assignment algorithm is specifically described in Algorithm 2. Since the subchannel assignments of different BSs are independent in this stage, the algorithm will complete the subchannel assignment of all BSs in parallel. For BS *m*, each user first sends a request to BS *m* to occupy the subchannel top-ranked in its preference list. On the BS side, after receiving the requests of users, it sorts all users on each subchannel according to (17) to generate $PL_{RA}^{mn}$. Finally, BS *m* will determine the subchannel assignment according to the requests and preference lists of subchannels.
**Algorithm 2 **Subchannel assignment based on matching algorithm.**Initialization**: $K_{m},M,S_{m},K_{m}^{rej}=K_{m},K_{mn}^{req}=K_{mn}^{rej}=\emptyset$.**for **m∈M** do**   **for** $k\in K_{m}$ **do**     User *k* generates its preference list $PL_{RA}^{mk}$.   **end for**   **while** $K_{m}^{rej}\neq \emptyset$ **do**     Update $K_{m}^{rej}\leftarrow K_{m}$     **for** $k\in K_{m}$ **do**        Find $n=\arg\max_{n\in PL_{RA}^{mk}}u_{RA}^{mk}\left(n\right)$.        Update $K_{mn}^{req}\leftarrow K_{mn}^{req}\cup \left\{k\right\}$.     **end for**     **for** $n\in S_{m}$ **do**        Subchannel *n* generates its preference list $PL_{RA}^{mn}$.        Find $k^{*}\in K_{mn}^{req}$ top-ranked in $PL_{RA}^{mn}$.        Allocate subchannel *n* to user $k^{*}$.        Update $K_{mn}^{rej}\leftarrow K_{mn}^{req}-\left\{k^{*}\right\}$.        User $k\in K_{mn}^{rej}$ removes subchannel *n* from $PL_{RA}^{mk}$.        Update $K_{m}^{rej}\leftarrow K_{m}^{rej}-\left\{k^{*}\right\}$.     **end for**   **end while****end for**

### 3.3. Power Allocation Optimization

To solve the power allocation problem, both the communication rate requirements of users and the interference in the HWN need to be considered. Since different subchannels do not interfere mutually, the power allocation on different subchannels is independent. Similar to the subchannel assignment problem, we divide the primal power allocation problem into *N* subproblems, each of which can be expressed as

(19) $$\begin{array}{cc} P1-{\mathrm{PA}}_{n}\; & \max_{P\left(\mu_{n}\right)}\;\sum_{\left(k,m\right)\in \mu_{n}}R_{k,m,n},\\ s.t.\; & R_{k,m,n}\geq R_{k}^{\min},\forall \left(k,m\right)\in \mu_{n},\\ \; & \sum_{\left(k,m\right)\in \mu_{n}}y_{k,m,n}p_{k,n}h_{k,0,n}\leq I_{0,n}^{th},\\ \; & p_{n}^{\min}\leq p_{k,n}\leq p_{n}^{\max},\forall \left(k,m\right)\in \mu_{n}. \end{array}$$

where $\mu_{n}$ denotes the set of user–BS pairs communicating on subchannel *n*.

The optimization problem (19) is a non-convex problem, which cannot be solved directly. We first convert the problem to a convex problem. When the SINR is high, it can be regarded as log(1+SINR)≈log(SINR). In the considered system, we assume that the SINR of the received signal is sufficiently high. Then, we have $R_{k,m,n}\approx B_{n}\log\left(\gamma_{k,m,n}\right)$. In addition, the SINR $\gamma_{k,m,n}$ contains the variable $p_{k,n}$ in both the numerator and denominator, leading to difficulties in solving the optimization problem. Therefore, we introduce the variable ${\bar{p}}_{k,n}=\ln\left(p_{k,n}\right)$ to replace $p_{k,n}$ in optimization problem (19). In addition, we define the variable $D_{k,m,n}≜\sum_{j\in K_{n},j\neq k}p_{j,n}h\left(j,m,n\right)$ to simplify the expression of mutual interference between BSs in the equation of $\gamma_{k,m,n}$. To facilitate the solution, the auxiliary variable ${\bar{D}}_{k,m,n}=\ln\left(D_{k,m,n}\right)$ is also introduced. Accordingly, the optimization problem (19) can be represented as

(20) $$\begin{array}{cc} P1-{\mathrm{PA}}_{n}^{\prime }\; & \min_{\bar{P},\bar{D}}\;\sum_{\left(k,m\right)\in \mu_{n}}\log\left[\frac{e^{-{\bar{p}}_{k,n}}}{h_{k,m,n}}\left(e^{{\bar{D}}_{k,m,n}}+h_{l,m,n}p_{l,n}+\sigma^{2}\right)\right],\\ s.t.\; & \log\left[\frac{e^{-{\bar{p}}_{k,n}}}{h_{k,m,n}}\left(e^{{\bar{D}}_{k,m,n}}+h_{l,m,n}p_{l,n}+\sigma^{2}\right)\right]+\frac{R_{k}^{min}}{B_{n}}\leq 0,\forall \left(k,m\right)\in \mu_{n},\\ \; & \sum_{\left(k,m\right)\in \mu_{n}}e^{{\bar{p}}_{k,n}}h_{k,0,n}-I_{0,n}^{th}\leq 0,\\ \; & \ln\left(p_{n}^{\min}\right)\leq {\bar{p}}_{k,n}\leq \ln\left(p_{n}^{\max}\right),\forall \left(k,m\right)\in \mu_{n}.\\ \; & {\bar{D}}_{k,m,n}=\ln\left(D_{k,m,n}\right),\forall \left(k,m\right)\in \mu_{n}. \end{array}$$

where $\bar{P}$ and $\bar{D}$ represent the sets of ${\bar{p}}_{k,n}$ and ${\bar{D}}_{k,m,n}$, $\forall \left(k,m\right)\in \mu_{n}$, respectively.

The optimization problem (20) is a convex problem that can be solved by using the Lagrange dual method [33,34,35]. First, we introduce the Lagrange multipliers $\lambda_{n}$, $\kappa_{k,m,n}$, and $\nu_{k,m,n}$, and then the Lagrange equation can be expressed as

(21) $$\begin{array}{cc} L({\bar{p}}_{k,n},{\bar{D}}_{k,m,n},\lambda ,\kappa ,\nu )=\; & \sum_{\left(k,m\right)\in \mu_{n}}\log\left[\frac{e^{-{\bar{p}}_{k,n}}}{h_{k,m,n}}\left(e^{{\bar{D}}_{k,m,n}}+h_{l,m,n}p_{l,n}+\sigma^{2}\right)\right]\\ \; & -\lambda_{n}\left(\sum_{\left(k,m\right)\in \mu_{n}}e^{{\bar{p}}_{k,n}}h_{k,0,n}-I_{0,n}^{th}\right)-\sum_{\left(k,m\right)\in \mu_{n}}\nu_{k,m,n}\left({\bar{D}}_{k,m,n}-\ln\left(D_{k,m,n}\right)\right)\\ \; & -\sum_{\left(k,m\right)\in \mu_{n}}\kappa_{k,m,n}\left(\log\left[\frac{e^{-{\bar{p}}_{k,n}}}{h_{k,m,n}}\left(e^{{\bar{D}}_{k,m,n}}+h_{l,m,n}p_{l,n}+\sigma^{2}\right)\right]+\frac{R_{k}^{min}}{B_{n}}\right). \end{array}$$

Using the Karush–Kuhn–Tucker (KKT) conditions, the optimal solution of (21) can be derived

(22) $$p_{k,n}^{*}={\left[\frac{1+\kappa_{k,m,n}}{\lambda_{n}h_{k,0,n}\ln\left(2\right)}\right]}^{P_{n}},\forall \left(k,m\right)\in \mu_{n},$$

where ${\left[a\right]}^{P_{n}}$ denotes that *a* is within $\left[p_{n}^{\min},p_{n}^{max}\right]$. In addition, the auxiliary variable $D_{k,m,n}$ can be expressed as

(23) $$D_{k,m,n}=e^{{\bar{D}}_{k,m,n}}={\left[\frac{\left(h_{l,m,n}p_{l,n}+\sigma^{2}\right)\nu_{k,m,n}\ln\left(2\right)}{1+\kappa_{k,m,n}-\nu_{k,m,n}\ln\left(2\right)}\right]}^{+},$$

where ${\left[a\right]}^{+}=\max\left\{a,0\right\}$.

Using the subgradient method, the Lagrange multipliers are iteratively updated until convergence, which are calculated as follows

(24) $$\lambda_{n}^{l+1}={\left[\lambda_{n}^{l}+\xi \left(\sum_{k\in \mu_{n}}e^{{\bar{p}}_{k,n}}h_{k,0,n}-I_{0,n}^{th}\right)\right]}^{+},$$

(25) $$\kappa_{k,m,n}^{l+1}={\left[\kappa_{k,m,n}^{l}+\zeta \log\left(\psi_{k,m,n}\right))+\frac{R_{k}^{min}}{B_{n}}\right]}^{+},$$

(26) $$\nu_{k,m,n}^{l+1}=\nu_{k,m,n}^{l}+\vartheta \left(D_{k,m,n}-e^{{\bar{D}}_{k,m,n}}\right),$$

where $\psi_{k,m,n}=\frac{e^{-{\bar{p}}_{k,n}}}{h_{k,m,n}}\left(e^{{\bar{D}}_{k,m,n}}+h_{l,m,n}p_{l,n}+\sigma^{2}\right)$, ξ, ζ, and ϑ are the update steps of the Lagrangian factors, and *l* denotes the number of iterations. In conclusion, Algorithm 3 describes the specific solution process of the power allocation problem.
**Algorithm 3 **Power allocation algorithm based on Lagrange dual method.**Initialization**: $S,\mu_{n},\bar{P}=\bar{D}=0$.**for **n∈S** do**   **for** $\left(k,m\right)\in \mu_{n}$ **do**     Estimate $D_{k,m,n}$.     **repeat**        Calculate $D_{k,m,n}^{l+1}$ according to Formula (23).        Calculate $\kappa_{k,m,n}^{l+1}$ according to Formula (25).        Calculate $\nu_{k,m,n}^{l+1}$ according to Formula (26).     **until** $D_{k,m,n},\kappa_{k,m,n},\nu_{k,m,n}$ converge.   **end for**   **for** $\left(k,m\right)\in \mu_{n}$ **do**     Estimate $p_{k,n}^{*}$.     **repeat**        Calculate $\lambda_{n}^{l+1}$ according to Formula (24).        Calculate $p_{k,n}^{l+1}$ according to Formula (22).     **until** $\lambda_{n},p_{k,n}$ converge.   **end for****end for**

### 3.4. Discussion

The proposed algorithm can reduce the computational complexity while the theoretically optimal solution cannot be guaranteed compared with the baselines. Specifically, the computational complexity mainly comes from three parts: user association optimization, subchannel assignment optimization, and power allocation optimization. The proposed algorithm can significantly reduce the computational complexity of the first two parts. Specifically, user association and subchannel assignment are integer programming problems, and they can be generally solved with exponential complexity while the matching-based algorithm can achieve polynomial complexity [36,37]. In terms of the third part, the Lagrange dual method adopted in our work also has polynomial complexity [38,39]. Therefore, the proposed algorithm can be effectively applied in practical systems to achieve acceptable performance with low complexity.

## 4. Simulation Results and Analysis

### 4.1. Simulation Setup

To verify the effectiveness of the proposed algorithm, we consider a 10 km ×10 km power monitoring scenario. A macro BS is deployed in the center of the area to provide signal coverage for the surrounding area of 5 km. In addition, in order to meet the communication demands of multiple terminals and further improve the system capacity, four UAVs equipped with micro BSs are deployed in the area to provide communication services for users in the surrounding area of 2 km. There are 28 power terminals randomly distributed in the area. Consider that each BS has eight subchannels available to users, each with a bandwidth of 1 MHz and a carrier frequency of 2 GHz. The macro BS provides communication services for eight users, and the remaining 20 users are allocated to the micro BSs. To balance the load of BSs, each micro BS is set to serve up to five users at the same time, that is, $q_{m}=5$. In addition, the maximum interference allowed for each subchannel of the macro BS is set to be −75 dBm, the noise power is set to be −114 dBm, the minimum communication rate of each user is set to be 2 Mbps, the maximum signal transmission power is set to be 1 W, and the signal transmission power of macro BS users is set to be 1 W.

Considering the power communication environment, we use the log-distance model of the macro cell to estimate the path loss, that is, $PL=128.1+37.6\log_{10}\left(d\right)$, where *d* represents the distance between the transmitter and receiver [40]. In addition, the Rayleigh fading model is used to estimate the small-scale fading. Then, the channel gain $h_{k,m,n}$ of user *k* to BS *m* on subchannel *n* can be represented as

(27) $$h_{k,m,n}=10^{(-128.1-37.6\log_{10}d_{k,m}+Raylrnd\left(B\right))/10},$$

where the standard deviation of the Rayleigh fading is set to be B=1 in the simulation.

### 4.2. Numerical Evaluation

**Figure 3:**
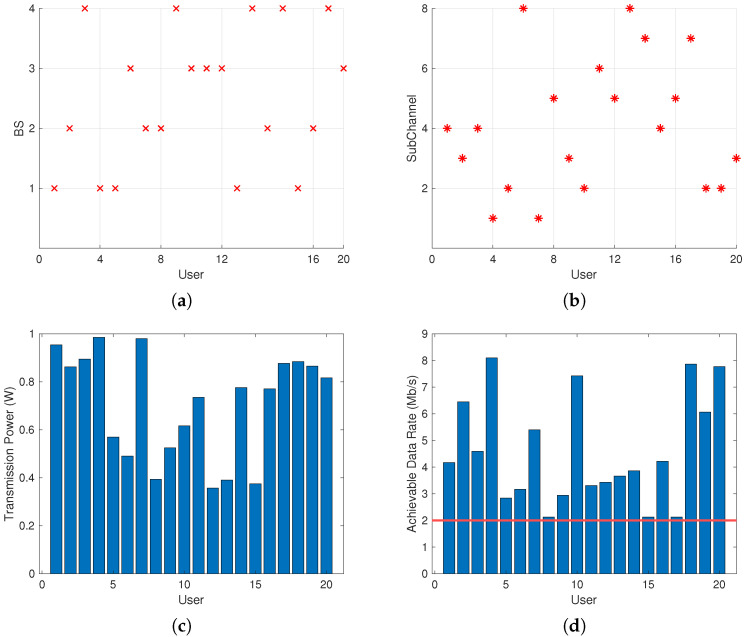
User association, subchannel assignment, power allocation, and user achievable data rate optimization results. (**a**) User association results. (**b**) Subchannel allocation results. (**c**) Power allocation results. (**d**) Achievable data rate of users.

Figure 3 depicts the execution results of the proposed algorithm. Figure 3a shows the connection between micro BSs and users. It can be seen that according to the matching method proposed in Algorithm 1, 20 users respectively establish connections with four micro BSs. Meanwhile, due to the setting of the maximum capacity of micro BSs, the four micro BSs provide communication services for five users, respectively, achieving the load balancing. Figure 3b shows the subchannel assignment result, and eight subchannels are assigned to 20 users, respectively. The subchannel assignment algorithm optimizes the user rate while tending to minimize the interference between users. Figure 3c demonstrates the power allocation result of the micro BS users, which is achieved with comprehensive consideration of the intra-layer interference, inter-layer interference, the user rate requirements, etc. Allocating transmission power to users according to the proposed algorithm can meet the various requirements of the entire communication system of the SG. Figure 3d illustrates the achievable data rate of micro BS users based on the proposed algorithm, where the red line represents the minimum required rate. It can be observed that the communication rate of each user in the SG can meet its minimum requirement, indicating the effectiveness of the proposed algorithm.

Furthermore, to fully evaluate the strengths and potential limitations of the proposed method, the proposed algorithm is simulated and compared with other five schemes, which are described as follows.

- Exhaustive search algorithm: An exhaustive method is adopted to obtain the optimal user association and subchannel assignment as in [41,42].
- Max-SINR algorithm: The max-SINR is adopted for user association as in [43], and the proposed Algorithm 2 is still used for subchannel assignment.
- Hungarian algorithm: The Hungarian algorithm is adopted for user association and subchannel assignment to find the maximum match as in [44].
- K-means algorithm: The K-means algorithm is adopted for user association as in [30,45], and the proposed Algorithm 2 is still used for subchannel assignment.
- Best channel gain algorithm: Users select the BS and subchannel with the best channel gain as in [46].

Note that the above schemes all use the proposed power allocation in Algorithm 3.

Under the power monitoring scenario described in Section 4.1, i.e., K=20, M=4, N=8, $p_{max}=1$, the comparison of the total system throughput and complexity of different schemes is shown in Table 1. From this table, the performance of the proposed algorithm is close to the exhaustive search algorithm. Meanwhile, compared to the exponential complexity of the exhaustive search algorithm, the proposed algorithm is able to solve the optimization problem in polynomial time. This indicates that the proposed algorithm achieves a great balance between computational complexity and performance. In addition, the system throughput of the proposed algorithm is 5.40% higher than that of the max-SINR algorithm, 2.76% higher than that of the Hungarian algorithm, 11.23% higher than that of the K-means algorithm, and 7.35% higher than that of the best channel gain algorithm.

**Figure 4:**
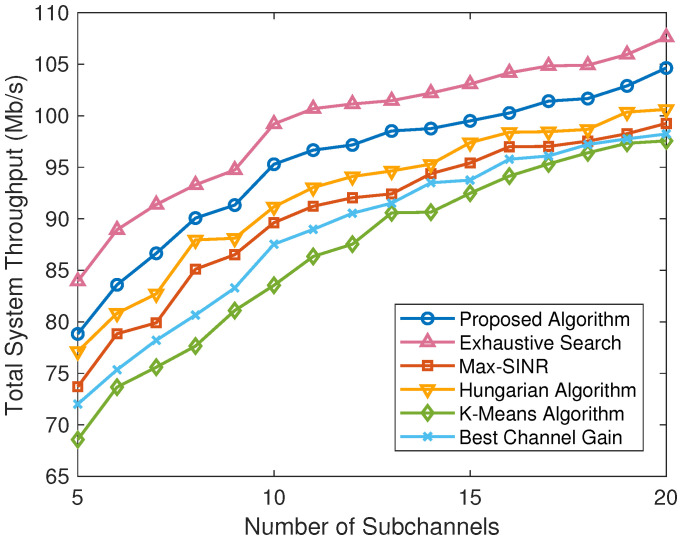
Total system throughput comparison of different algorithms with different numbers of users.

**Figure 5:**
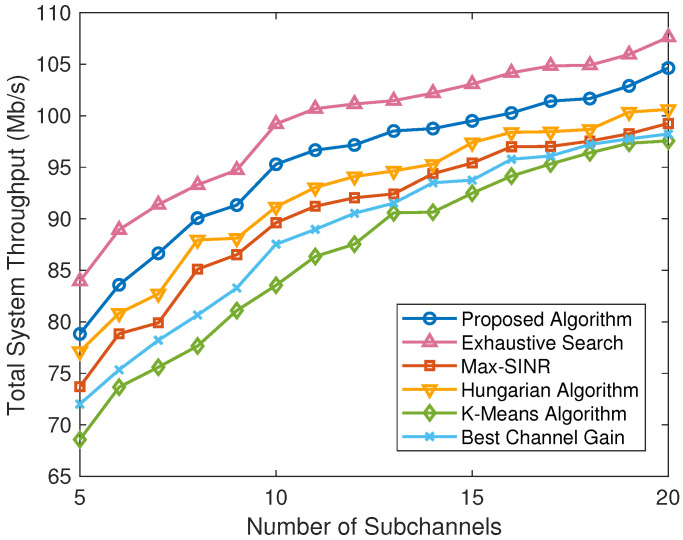
Total system throughput comparison of different algorithms with different numbers of subchannels.

**Figure 6:**
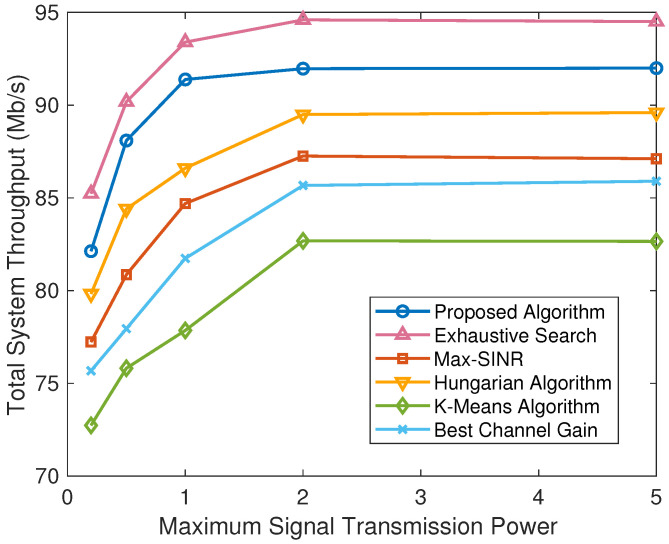
Total system throughput comparison of different algorithms with different maximum signal transmission power.

To further compare the different schemes under various scenarios, we obtain the simulation results under the varying number of users, the varying number of available subchannels, and the varying maximum transmission power in Figure 4, Figure 5, and Figure 6, respectively.

Figure 4 shows the total system performance comparison under different numbers of users. It can be seen that the total system throughput of both the proposed algorithm and the other five schemes gradually increases as the number of users increases. When the number of users reaches the maximum capacity, the system throughput remains stable. This is because the number of user connections of all BSs in the system reaches the limit. Also, the system throughput achieved by the proposed algorithm is always superior to that of the max-SINR algorithm, the Hungarian algorithm, the K-means algorithm, and the best channel gain algorithm.

Figure 5 describes the total system performance comparison between the proposed algorithm and five baselines with different numbers of available subchannels. It can be seen that the total system throughput of different algorithms initially increases with the increase of the number of system subchannels. This is because when the number of available subchannels is relatively small, there will be multiple users sharing a single subchannel, and the interference between users is severe. In this case, the transmission power of the users under the limit of the interference threshold is small, so the system throughput is low. When the number of available subchannels is large enough, different users can occupy different subchannels, or even each user can occupy a completely independent subchannel. Therefore, the achievable data rate of the users increases due to there being no interference between these users. It can also be seen that the performance of the proposed algorithm is significantly better than the max-SINR algorithm, the Hungarian algorithm, the K-means algorithm, and the best channel gain algorithm, which is close to the upper limit provided by the exhaustive search algorithm, demonstrating the effectiveness of the proposed algorithm.

Figure 6 depicts the total system performance comparison between the proposed algorithm and five baselines under different maximum transmission power. It can be seen that with the increase in the user transmission power, the total system throughput of all four schemes gradually increases and finally reaches stability. This is because when the transmission power increases to a certain degree, the interference to BSs will be larger, and then the proposed power allocation algorithm will limit the transmission power of users to avoid excessive interference. There is an upper limit to the total system throughput as the transmission power increases. In addition, the exhaustive search algorithm in Figure 6 can also achieve the highest performance, while a similar performance can be obtained by the proposed algorithm with a low complexity.

According to the simulation results in Figure 4, Figure 5, and Figure 6, our proposed method achieves acceptable performance compared to baselines in different scenarios, such as different numbers of users and different amounts of communication resources. Although the performance is lower than the exhaustive search algorithm, it has the advantage of lower complexity, which is more appropriate for practical systems.

## 5. Conclusions

In this paper, to meet the demands on the reliable communication in the SG, especially in remote areas, a UAV-assisted HWN architecture is proposed. To further improve the capacity of the smart grid communication system, an efficient algorithm is developed to maximize the system throughput by jointly adjusting the user association and resource allocation. Herein, the constraints on the intra-layer and inter-layer interference of the HWN, the communication demand of users, and the load balancing of the BSs are taken into account. To solve this non-convex optimization problem, we first decompose the original optimization problem into three subproblems: a user association problem, subchannel assignment problem, and power allocation problem. For the user association and subchannel assignment problems, a matching algorithm is designed to accomplish the matching between users and base stations as well as between users and subchannels. For the power allocation problem, we use the Lagrangian dual method to obtain the closed-form solution of power allocation. Finally, the proposed algorithm is validated by simulation. The simulation results show that the proposed algorithm can enhance the system capacity while satisfying the user communication requirements in the SG. In addition, it has the performance close to the exhaustive search algorithm, and it outperforms the max-SINR algorithm, the Hungarian algorithm, the K-means algorithm, and the best channel gain algorithm. Future research will focus on several key challenges in UAV-assisted communications, especially UAV power management and trajectory optimization.

## Figures and Tables

**Table 1:** Comparison of the total system throughput and complexity of different schemes.

Schemes	Total System Throughput (Mb/s)	Complexity
Proposed Algorithm	91.969	Medium
Exhaustive Search Algorithm [41,42]	94.599	High
Max-SINR Algorithm [43]	87.256	Medium
Hungarian Algorithm [44]	89.495	Medium
K-means Algorithm [30,45]	82.682	Low
Best Channel Gain Algorithm [46]	85.670	Low

## Data Availability

The data generated during and/or analyzed during the current study are available from the corresponding author upon reasonable request.
